# Integrated Proteomic Analysis of Alzheimer and Mild Cognitive Impairment in Biofluid using Mass Spectrometry and NULISA^TM^ Technology

**DOI:** 10.1002/alz.091340

**Published:** 2025-01-09

**Authors:** Yao Chen, Hendrik Wesseling, Mikhail Levit, Bailin Zhang

**Affiliations:** ^1^ Sanofi, Cambridge, MA USA

## Abstract

**Background:**

One hypothesis of lack of effectiveness in most current clinical trials for the AD (Alzheimer’s disease) is the advanced stages of the disease at the time of pharmacological intervention. To aim a robust effect from disease‐modifying therapies, there is an urgent need for biomarkers that can identify patients with MCI and early stages of AD. In this study, we combined in‐house large‐scale LC‐MS and new emerging highly sensitive NULISA proteomics analysis on a medium sized AD cohort sample, aiming an early‐stage protein biomarker discovery in MCI and AD.

**Method:**

59 AD set plasma and 65 AD set CSF were purchased from the vendor. Donor classification strictly followed the disease inclusion/exclusion criteria. One aliquot from each sample were sent to Alamar Bioscience for NULISA_seq_ analysis on ARGO^TM^ system using 120‐plex CNS disease panel. In house LC‐MS was performed on the same set of samples.

**Result:**

Of 59 AD set plasma samples (19 HC, 22 MCI AND 18 AD), 3 significantly up‐regulated protein in MCI and 16 significantly down‐regulated protein in AD and MCI were identified by NULISA_seq_ 120plex CNS panel. One significantly up‐regulated protein in MCI and 3 significantly down‐regulated proteins in AD were also confirmed by in house LC‐MS. Of 65 AD set CSF samples (19 HC, 22 MCI and 24 AD), 12 significantly up‐regulated proteins and 11 significant down‐regulated protein in AD and MCI were identified by NULISA_seq_ 120plex CNS panel. CSF protein profiling by in house LC‐MS is still pending. 5 (3 from plasma, and 2 from CSF) significantly up‐regulated novel proteins in MCI and CD have been reported to be associated with neuronal connectivity, neuro inflammation or acting as a neuromodulator. Sample correlation in CSF between NULISA_seq_ and Simoa (Single Molecule Array) was also verified by NFL and pTau181. Both proteins displayed excellent correlation (R^2^ was 0.88 and 0.92).

**Conclusion:**

5 novel significantly upregulated protein biomarkers in MCI and AD were identified by combined in house MS and new emerging NULISA_seq_ technologies. The integrated proteomic method could be a powerful tool to identify the molecular signature and pathological pathways associated with AD and MCI.

